# A LigA Three-Domain Region Protects Hamsters from Lethal Infection by *Leptospira interrogans*

**DOI:** 10.1371/journal.pntd.0001422

**Published:** 2011-12-13

**Authors:** Mariana L. Coutinho, Henry A. Choy, Melissa M. Kelley, James Matsunaga, Jane T. Babbitt, Michael S. Lewis, Jose Antonio G. Aleixo, David A. Haake

**Affiliations:** 1 Veterans Affairs Greater Los Angeles Healthcare System, Los Angeles, California, United States of America; 2 Centro de Desenvolvimento Tecnologico, Universidade Federal de Pelotas, Pelotas, Brasil; 3 Department of Medicine, David Geffen School of Medicine at University of California Los Angeles (UCLA), Los Angeles, California, United States of America; 4 Department of Urology, David Geffen School of Medicine at UCLA, Los Angeles, California, United States of America; 5 Department of Microbiology, Immunology and Molecular Genetics, UCLA, Los Angeles, California, United States of America; University of Washington, United States of America

## Abstract

The leptospiral LigA protein consists of 13 bacterial immunoglobulin-like (Big) domains and is the only purified recombinant subunit vaccine that has been demonstrated to protect against lethal challenge by a clinical isolate of *Leptospira interrogans* in the hamster model of leptospirosis. We determined the minimum number and location of LigA domains required for immunoprotection. Immunization with domains 11 and 12 was found to be required but insufficient for protection. Inclusion of a third domain, either 10 or 13, was required for 100% survival after intraperitoneal challenge with *Leptospira interrogans* serovar Copenhageni strain Fiocruz L1-130. As in previous studies, survivors had renal colonization; here, we quantitated the leptospiral burden by qPCR to be 1.2×10^3^ to 8×10^5^ copies of leptospiral DNA per microgram of kidney DNA. Although renal histopathology in survivors revealed tubulointerstitial changes indicating an inflammatory response to the infection, blood chemistry analysis indicated that renal function was normal. These studies define the Big domains of LigA that account for its vaccine efficacy and highlight the need for additional strategies to achieve sterilizing immunity to protect the mammalian host from leptospiral infection and its consequences.

## Author Summary

Leptospirosis is the most widespread bacterial infection transmitted to humans from host animals that harbor the bacteria in their kidneys. Human infections caused by the bacterium, *Leptospira interrogans*, frequently result in a life-threatening illness characterized by jaundice and kidney failure. Vaccines are urgently needed to prevent leptospirosis in populations at risk. The leptospiral protein, LigA, is a promising vaccine candidate because it is the first purified protein to be shown to protect animals from fatal leptospirosis. The goal of this study was to determine which of LigA's 13 domains are required for the protective effect. Immunization with domains 11 and 12 was found to be required, but was insufficient, for protection. A third domain, either 10 or 13, was required for 100% survival. As in previous studies, residual bacteria were cultured from the kidneys of survivors. However, in contrast to previous studies, we determined the amount of bacterial DNA in the kidneys as a measure of vaccine efficacy. We also examined the kidneys microscopically for signs of damage and measured blood chemistries to assess kidney function. These are important steps towards developing vaccines that provide protection from kidney damage and infection.

## Introduction

Pathogenic *Leptospira* species are globally distributed spirochetes that cause 350,000–500,000 severe human infections annually with an incidence of >10 cases per 100,000 population in humid, subtropical regions of the world and a mortality rate of 10% [1], [2], [3]. These figures are likely to be underestimates because leptospirosis is a neglected tropical disease that occurs more commonly among medically underserved populations [4], [5]. The infection is endemic wherever there is exposure to urine of reservoir host animals that harbor the organism in their renal tubules [6]. At least 18 species and more than 200 leptospiral serovars have been described, many of which were isolated by cultivation of kidneys from a wide diversity of infested wild and domestic animals [1], [7]. Environmental contamination of water and soil results in frequent outbreaks of leptospirosis among the poor in developing countries. Leptospirosis is also emerging among participants of aquatic sports and adventure tourism [8], [9]. In the urban setting, *Rattus norvegicus* is the most important vector of human leptospirosis [5]. Serovars of *Leptospira interrogans* carried by rats cause life-threatening hepatorenal failure and pulmonary hemorrhage syndromes in tropical regions, especially where heavy rainfall occurs in urban areas with poor sanitation and flood control infrastructure [10]. Commercially available whole-cell bacterin vaccines for prevention of leptospirosis in animals provide relatively short-term serovar-specific protection and require frequent boosters [11]. Although inactivated whole-cell vaccines have been administered to humans, they are rarely used today because of their reactogenicity. Thus, there is an urgent need for development of novel vaccine strategies that provide safe, long-term, cross-protective immunity.

Recombinant surface-exposed outer membrane proteins (OMPs) are attractive subunit vaccine candidates because in contrast to the lipopolysacchride, leptospiral OMPs are relatively well conserved and those that are surface-exposed represent potential targets for immune-mediated defense mechanisms. We have developed a suite of complementary approaches for determining which leptospiral OMPs are surface-exposed, including surface immunofluorescence, surface biotinylation, surface proteolysis, surface immunoprecipitation, and surface ELISA [12], [13], [14], [15]. Using these approaches, a number of transmembrane OMPs and surface lipoproteins have been identified [16], [17]. Despite the rapid increase in knowledge about leptospiral OMPs, progress in understanding their vaccine potential has been slow. Although LipL32 is the most abundant pathogenic leptospiral OMP [18], purified, recombinant LipL32 has no detectable vaccine efficacy [19]. Nevertheless, hamsters immunized with recombinant bacillus Calmette-Guerin expressing LipL32 were partially protected from lethal challenge [20] and there is evidence for immunoprotection employing *lipL32*-containing viral or DNA-based vectors [21], [22]. Synergistic immunoprotection has been observed using a combination of leptospiral OMPs, OmpL1 and lipidated LipL41, expressed as membrane proteins in *E. coli* [23].

Leptospiral immunoglobulin-like (Lig) proteins are of great interest as mediators of leptospiral pathogenetic mechanisms, as serodiagnostic antigens, and as effective recombinant vaccinogens [24], [25], [26], [27], [28]. At least two of the three members of the Lig protein family are outer membrane lipoproteins containing a tandem series of bacterial immunoglobulin-like (Big) domains [29]. Lig protein expression is associated with virulence and is strongly and rapidly induced by increasing the osmolarity of the culture medium to physiologic levels found in the mammalian host, suggesting that they may be involved in the initial stages of host tissue colonization [30], [31]. LigA consists of 13 Big domains, the first six of which are nearly identical in sequence to those in LigB, while the last seven are unique to LigA [32] and mediate interactions with host extracellular matrix proteins and fibrinogen [24], [33]. One study has found that the region shared by LigA and LigB was not immunoprotective [27], while another study reported that this region conferred some immunoprotective activity [34]. In contrast, several groups have reported that immunization with the LigA-unique region induced protection from lethal infection either in a mouse model [28] or in the hamster model [27], [35] of leptospirosis. Although hamsters surviving leptospiral challenge were found to have sublethal kidney infection, both the extent of infection and its effects on the kidney, the key target organ in leptospirosis, were not well understood. In this study, we determined which LigA domains are most strongly associated with immunoprotection and the effect of LigA immunization on the burden of infection and the histopathology in the kidney. Our results show that protection from lethal infection required immunization with domains 11 and 12 along with a third domain, either 10 or 13.

## Materials and Methods

### Leptospiral strain and cultivation

*L. interrogans* serovar Copenhageni strain Fiocruz L1-130 was maintained in Ellinghausen-McCullough-Johnson-Harris (EMJH) medium [36] supplemented with 1% rabbit serum (Rockland Immunochemicals, Gilbertsville, PA) and 100 µg/ml 5-fluorouracil at 30°C in a shaker incubator. Organisms were passaged no more than five times prior to hamster challenge. Hamster tissues were cultured in semi-solid EMJH or semi-solid Probumin Vaccine Grade Solution (Millipore, Billirica, MA) containing 0.2% Bacto agar (BD, Franklin Lakes, NJ) and 100 µg/ml 5-fluorouracil in a stationary incubator at 30°C and were examined for leptospiral growth for up to two months.

### Preparation of recombinant proteins

PCR primers were designed to amplify gene fragments encoding various immunoglobulin-like domains from *ligA* of *L. interrogans* serovar Copenhageni strain Fiocruz L1-130 (Table 1). DNA amplicons, which included *Nde* I and *Xho* I restriction endonuclease sites, were ligated into pET-20b(+) (Novagen), providing a carboxy-terminal His6 tag, and used to transform *Escherichia coli* BLR(DE3)pLysS (Novagen). Protein expression was induced with isopropyl-β-D-thiogalactopyranoside at 30°C and soluble proteins were released with BugBuster (Novagen) and purified with nickel-affinity chromatography as previously described [25]. All proteins were stored at 4°C after dialysis in PBS.

**Table 1:** Recombinant LigA proteins.

Protein^a^	Amino acidcoordinates^b^	MW (Da)	Primers^c^
**LigA7′-13**	L631-P1224	63,422	f-AACATATCTCATATGCTTACCGTTTCCAACACAAACGCCAAr-TTCCTCGAGTGGCTCCGTTTTAATAGAGGCTAAT
**LigA7′-11**	L631-A1033	42,991	f-AACATATCTCATATGCTTACCGTTTCCAACACAAACGCCAAr-GACGTCCTCGAGAGCAGAAGTGACATACAAGGTAGTAGA
**LigA7′-9**	L631-A851	24,034	f-AACATATCTCATATGCTTACCGTTTCCAACACAAACGCCAAr-AGTCTCGAGCGCTGCGGTAACGGATAATTTGGA
**LigA10-13**	E852-P1224	40,602	f-ACGCTTACGCATATGGAACTTACTGAGATTGTGCTAAATCCr-TTCCTCGAGTGGCTCCGTTTTAATAGAGGCTAAT
**LigA10-12**	E852-E1124	30,085	f-ACGCTTACGCATATGGAACTTACTGAGATTGTGCTAAATCCr-GCGTAGCTCGAGCTCGTCATTGACGAATATCCA
**LigA11-13**	R943-P1224	31,233	f-CATCAATGACATATGAGAATAGCTTCAATCGAAGTAACACCr-TTCCTCGAGTGGCTCCGTTTTAATAGAGGCTAAT
**LigA11-12**	R943-E1124	20,716	f-CATCAATGACATATGAGAATAGCTTCAATCGAAGTAACACCr-GCGTAGCTCGAGCTCGTCATTGACGAATATCCA
**LigA12-13**	V1034-P1224	21,645	f-ATACAGTCTCATATGGTCCTTATTGACATAGAAGTCAAGCCr-TTCCTCGAGTGGCTCCGTTTTAATAGAGGCTAAT

a LigA protein designations list the first and last domains included in the construct; 7′ indicates a half domain.

b Coordinates refer to the first and last amino acids in the LigA protein of *L. interrogans* serovar Copenhageni strain Fiocruz L1-130.

c Forward (f) and reverse (r) primer sequences, including an *Nde* I or *Xho* I site, respectively, are listed in the 5′ to 3′ direction.

### Hamster immunization

Groups of four female Syrian hamsters, 5 to 6 weeks of age (Harlan Bioscience, Indianapolis, IN), were immunized subcutaneously with 100 µg of recombinant protein, PBS, or 1×10^8^ heat-killed (56°C for 1 h) leptospires (HKL) in a total volume of 0.5 mL on days 0, 14 and 28 with Freünd's adjuvant (complete adjuvant for the first immunization, incomplete adjuvant for subsequent immunizations). Blood samples were obtained two days before the first immunization and 10 to 12 days after each immunization *via* the retro-orbital route. All animal procedures were approved by the Veterans Affairs Greater Los Angeles Healthcare System Institutional Animal Care and Use Committee and adhere to the United States Health Research Extension Act of 1985 (Public Law 99–158, November 20, 1985, “*Animals in Research*”), the National Institutes of Health's *Plan for Use of Animals in Research* (Public Law 103–43, June 10, 1993), U.S. Government Principles for the Utilization and Care of Veterbrate Animals Used in Testing, Research, and Training, Public Health Service Policy on Humane Care and Use of Laboratory Animals, the United States Department of Agriculture's Animal Welfare Act & Regulations, and Veterans Health Administration Handbook 1200.7.

#### ELISA

Ninety-six-well ELISA microtiter plates (Immulon 4HBX,Thermo Fisher, Waltham, MA) were coated either with 100 µL of 10 µg/mL of recombinant LigA protein or 1×10^9^ heat-inactivated leptospires/mL diluted in PBS, pH7.2 (Invitrogen, Carlsbad, CA), by overnight incubation at 4°C. The plates were allowed to warm to room temperature (RT), washed once with 200 µL of PBS, and blocked with Protein-Free Blocking Buffer (PFBB, Thermo Fisher, Rockford, IL) for 1 to 2 h at RT. Wells were washed with PBS, sera diluted with PFBB were added in a volume of 100 µL, and plates were incubated for 1 h at 37°C. Non-binding antibodies were removed with three PBS washes, and Horseradish peroxidase (HRP)-conjugated anti-Syrian hamster immunoglobulin secondary antibody (Jackson ImmunoResearch, West Grove, PA) 1:5000 was incubated for 30 min at RT. Following three washes with PBS, 100 µL of 1-Step Turbo TMB HRP substrate (Thermo Fisher) was added and incubated for 30 min at RT with shaking. The reaction was stopped by the addition of 50 µL of 2 M H_2_SO_4_, and plates were immediately read in a Bio-Rad 550 Microplate Reader at 450 nm. End-point titers were defined as the highest titer that yielded a reading two standard deviations above the result with sera from PBS-immunized hamsters. Geometric mean end-point titers were calculated as previously described [37].

### Challenge and sample collection

Fourteen days after the third immunization (day 42), hamsters were challenged intraperitoneally with 1×10^3^ *L. interrogans* serovar Copenhageni strain Fiocruz L1-130 in 0.5 mL of EMJH. The animals were weighed daily and observed for end-point criteria, including loss of appetite, gait or breathing difficulty, prostration, ruffled fur, or weight loss of ≥10% of the animal's maximum weight. Animals that reached end-point criteria were euthanized with isoflurane and tissue samples were collected in formalin for histopathology or incubated overnight at 4°C in RNAlater (Ambion, Austin, TX) and stored at −80°C. Processing tissues for histopathology involved formalin fixation, paraffin embedding, sectioning, and periodic acid Schiff (PAS) staining in a Dako automated slide processor. Blinded scoring of kidney sections used a scale of 0 to 5 for the extent of histopathology, ranging from normal to severe renal tubular damage, based on the degree of hyaline cast deposition, interstitial inflammation, mitosis, Bowman's space dilation, tubular atrophy and associated capsular depression. Blood was collected for serology and chemistry analysis (Antech Diagnostics, Irvine, CA). 100 µL of blood or pulverized kidney or liver were inoculated into semi-solid medium at dilutions of 1:100 and 1:10,000 and incubated at 30°C.

### Microscopic agglutination test (MAT)

Sera collected at euthanasia were examined at a 1:50 dilution by MAT as previously described [38] with live *L. interrogans* serovar Copenhageni strain Fiocruz L1-130. Briefly, heat-inactivated serum, diluted in physiologically buffered water, pH7.6, was incubated overnight at 4°C with 2 to 4×10^8^ leptospires/mL and examined under dark-field microscopy for >50% reduction in the number of free leptospires when compared with serum from uninfected animals.

### Quantitative PCR (qPCR)

Kidneys were stored in RNAlater and DNA was extracted with DNeasy Blood and Tissue kit according to the manufacturer instructions (Qiagen, Valencia, CA) with modifications. 15 to 25 mg of kidney were immersed in 360 µL of ATL buffer and the tissue was homogenized in a 24-Fast Prep tissue homogenizer (MP Biomedicals, Solon, OH) using lysing matrix A with a setting of 6 m/s for 40s. 40 µL of proteinase K at a concentration of 15 mg/mL of protein were added and the samples were incubated for 3 h at 37°C. Two volumes of AL buffer-ethanol (1:1) were added and the mixture was applied to a spin column, on which the bound DNA was washed with washing solutions 1 and 2 and eluted with 200 µL of AE buffer-water (1:4). The purified DNA was stored at −80°C until use.

The extracted DNA was used in a qPCR using the Bio-Rad iQ5 Real-time System (Bio-Rad, Hercules, CA). 100 ng of total DNA was combined with 1 µM of each primer and 12.5 µL iQ SYBR Green Supermix (Bio-Rad) and brought to a final volume of 25 µL with nuclease-free water (Ambion). 4 samples were run per group and each sample was run in duplicate. qPCR primer pairs were LipL32-f, 5-CGCGTTACCAGGGCTGCCTT-3′, and LipL32-r, 5′-CGCTTGTGGTGCTTTCGGTG-3′, and hamster GAPDH-f, 5′-CTGGTTACCAGGGCTGCCTT-3′, and GAPDH-r, 5′-CCGTTCTCAGCCTTGACTGTGC-3′, resulting in amplicons of 152 bp and 146 bp, respectively. The PCR protocol consisted of an initial incubation step at 95°C for 12.5 min followed by 40 cycles of amplification (95°C for 15 s, 57°C for 30 s and 72°C for 30 s). The level of the *lipL32* gene of *L. interrogans* was normalized to that of hamster *gapdh*, using Bio-Rad iQ5 software and Microsoft Excel. Standard curves were generated for each gene ranging from 10 to 1.6×10^6^ copies of *Leptospira* (20-fold dilutions) and 0.02 to 200 ng (10-fold dilutions) of hamster DNA.

### Statistics

Survival differences between groups were analyzed by Fisher's Exact Test using GraphPad InStat version 3.10 (GraphPad Software Inc., La Jolla, CA). One-way analysis of variance (ANOVA) was used to test for differences between multiple (≥3) groups using a P value<0.05. For ordinal data, such as the histopathology scores, the Kruskal-Wallis one-way ANOVA with Dunn's post-test was included. The unpaired, two-tailed Student's t-test assuming unequal variance was used to test for differences between two groups using a P value<0.05.

## Results

### Recombinant LigA proteins and hamster response to immunization

**Figure 1:**
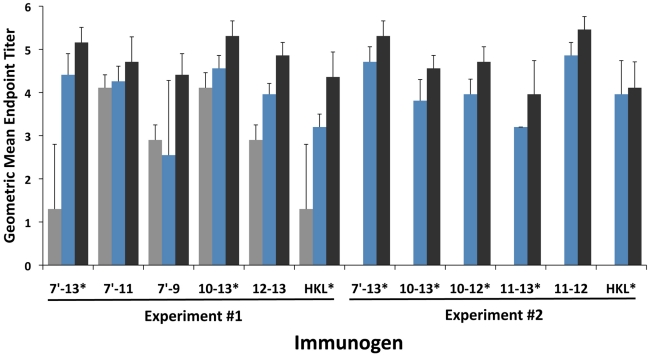
Antibody response in hamsters immunized with recombinant LigA proteins or heat-killed leptospires (HKL). Total hamster immunoglobulin responses to immunogens were measured by ELISA. Geometric mean end-point titers and standard deviations (n = 4) are shown after the first (gray), second (blue), and third (black) immunizations. In all cases, pre-immune sera were negative. Recombinant LigA proteins are represented by their domain numbers. Asterisks indicate proteins that provided 100% protection against lethal challenge.

Eight clones were designed to express recombinant proteins corresponding to various LigA domains from the second half of domain 7 to domain 13 (Table 1) of *L. interrogans* serovar Copenhageni. All proteins were expressed and purified as soluble proteins and found to be stable at 4°C after dialysis in sterile PBS. These proteins were employed as hamster immunogens in two independent experiments (#1 and #2) and as antigens in an indirect ELISA to measure the corresponding antibody response. As shown in Figure 1, hamsters had higher antibody titers after the third immunization than after one or two immunizations (one-way ANOVA with test for linear trend, P<0.05), except in the HKL (experiment #2) and LigA7′-11 groups. There was no correlation between the antibody titer and the number of domains in the LigA protein (Pearson correlation coefficient 0.29, P>0.05).

### Immunoprotective LigA domains

**Figure 2:**
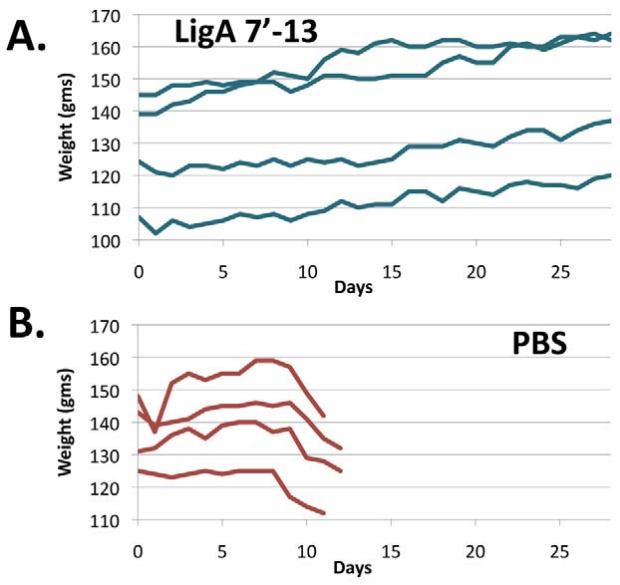
Hamster weight as an end-point for leptospiral infection. Animals were weighed at the time of challenge and daily thereafter for 28 days. Data are shown for experiment #2. **A.** Animals immunized with recombinant LigA7′-13 progressively gained weight (lines represent individual animals). **B.** Control animals sham-immunized with phosphate-buffered saline (PBS) had stable or increasing weights until day 8 or 9 after challenge, after which they lost weight and met the end-point criterion of a 10% weight decrease (lines represent individual animals).

Hamsters were challenged with virulent *L. interrogans* via the intraperitoneal route and observed daily, with a 10% decrease in body weight included as an end-point criterion. Body weight was found to be a useful measure of the response of animals to challenge; a decrease in body weight was the earliest observable sign of clinical leptospirosis. In contrast to animals that were immunized with LigA7′-13 and exhibited 100% challenge survival (Figure 2A, Table 2), non-surviving animals that were sham-immunized with PBS began to lose weight on day 8 after the challenge and reached -10% of peak weight within 48 h (Figure 2B).

**Table 2:** Summary of immunoprotection outcomes^a^.

Immunogen(LigA Domains)	Survival^b^(%)	MAT(Positives/Total)	Culture^c^(Positives/Total)	Histology^c^(Mean Score)	qPCRc,d(Log10)
Experiment 1					
7′-13	100	3/3	4/4	ND	5.79±2.0
7′-9	0*	1/3	4/4	ND	3.92±0.3
7′-11	50	2/4	2/4	ND	3.10**±0.8
10-13	100	4/4	4/4	ND	5.92±0.8
12-13	50	0/4	4/4	ND	5.53±1.3
HKL	100	3/3	0/4	ND	2.48**±0.6
PBS	0*	0/1	4/4	ND	4.66±1.0
Experiment 2					
7′-13	100	4/4	4/4	1.0	4.13±0.2
10-13	100	4/4	4/4	2.5	4.90±1.0
10-12	100	4/4	4/4	3.3**	4.81±0.4
11-13	100	4/4	4/4	1.75	5.61±1.2
11-12	25	4/4	1/4	3.3**	4.29±0.8
HKL	100	3/4	0/4	1.25	2.39**±0.2
PBS	0*	3/3	2/4	ND	2.72**± 0.2

a Abbreviations: HKL  =  heat-killed leptospires, PBS  =  phosphate-buffered saline, ND  =  not done.

b Four animals per group. *Statistically different from the LigA7′-13 group (Fisher's exact test, P<0.05).

c Data refer to kidney analysis. Means are shown for Histology and qPCR (n = 4).

**Statistically different from the LigA7′-13 group (ANOVA, Dunn post-test, P<0.05).

d Expressed as copies per microgram of tissue DNA.

**Figure 3:**
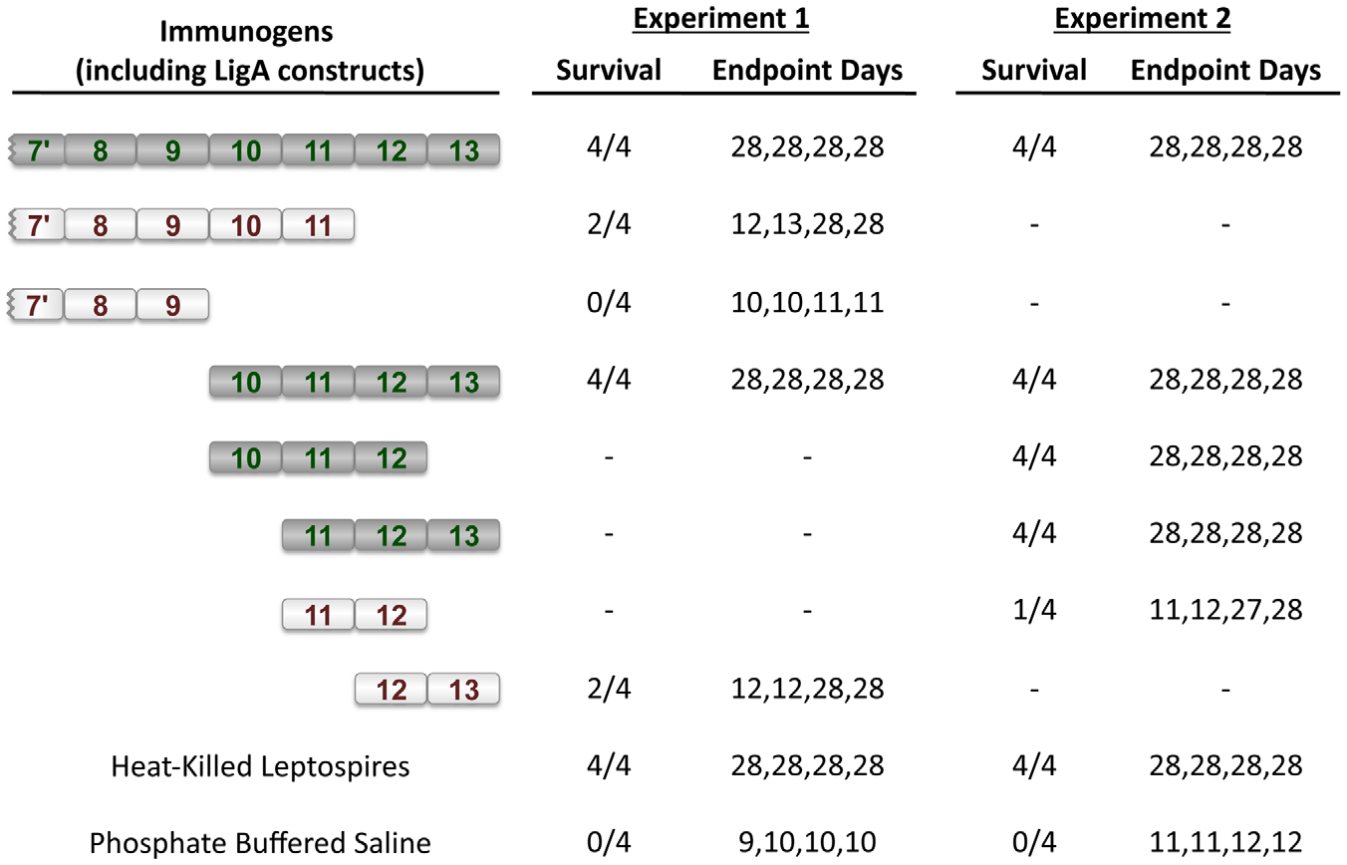
Mapping of the immunoprotective segment of LigA. Recombinant LigA proteins were tested for protective efficacy. The number of animals surviving (survivors/total) and days to endpoint after challenge are shown. Surviving animals were observed for up to 28 days. Ig-like domains of fully protective proteins are represented by dark symbols with green numbers while Ig-like domains of partially protective and non-protective proteins are represented by white symbols with red numbers.

Immunization with different recombinant LigA protein constructs (Table 1) resulted in dramatically different challenge outcomes (Table 2 and Figure 3). In both experiments, there was 100% survival in hamsters immunized with either the LigA7′-13 or LigA10–13 proteins. In experiment #1, immunization with either the LigA7′-11 protein or the LigA12-13 protein resulted in<50% survival. This result indicated that no single LigA domain was sufficient to afford 100% immunoprotection. For this reason, a second experiment was performed to identify the LigA domain(s) and the minimum number of domains required to protect hamsters from lethal challenge. Interestingly, both the LigA10-12 and the LigA11-13 proteins were both effective immunogens, while the LigA11-12 protein consisting of their shared domains afforded only 25% survival. Taken as a whole, these data indicate that LigA domains 11 and 12 are required but not sufficient to induce 100% survival. A recombinant LigA protein construct consisting of at least three specific Big domains is needed to induce a maximally protective immune response. The protective effect was not merely a reflection of antibody titer; as there was no correlation between survival and geometric mean end-point titer (Figure 1, one-way ANOVA, P > 0.05).

### Effect of LigA immunization on organ colonization

**Figure 4:**
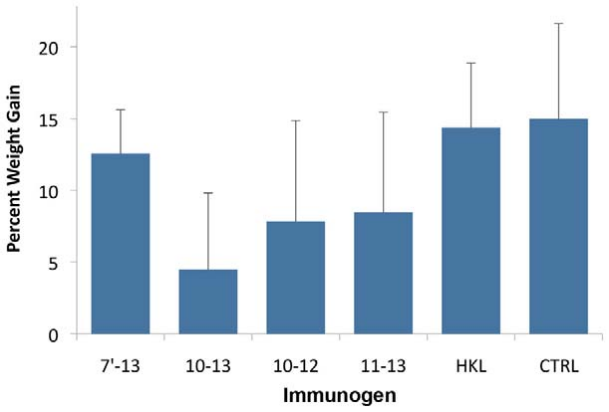
Percentage weight gain in hamsters immunized with protective immunogens. Mean and standard deviation (n = 4) of percent weight gain from challenge to 28 days in groups that had 100% survival, including hamsters immunized with recombinant LigA proteins (represented by their domain numbers), heat-killed leptospires (HKL) and a non-immunized and unchallenged control (CTRL) group.

As previously reported [27], immunization with LigA proteins provided non-sterilizing immunity, as organisms were isolated from the kidneys of animals surviving challenge. Cultures of kidney tissue from all hamsters surviving to 28 days were positive (Table 2). In contrast, only 3 and 10 of 56 animals had positive liver and blood cultures, respectively (data not shown). One non-surviving animal immunized with LigA11-12 had a positive blood culture but negative cultures of the kidney and liver. The residual kidney infection was reflected in lower weight gain of hamsters after challenge (Figure 4). Among the surviving hamsters, those immunized with LigA10-13 had a non-statistical trend of gaining less weight after challenge than those immunized with LigA7′-13 or heat-killed leptospires. Infection resulted in the formation of agglutinating antibodies; the MAT was positive in nearly all LigA-immunized animals surviving for 28 days (Table 2). The only exceptions were one animal from the HKL control group and two from the LigA12-13 group that met end-point criteria early, the latter presumably because these animals had insufficient time to develop agglutinating antibodies.

**Figure 5:**
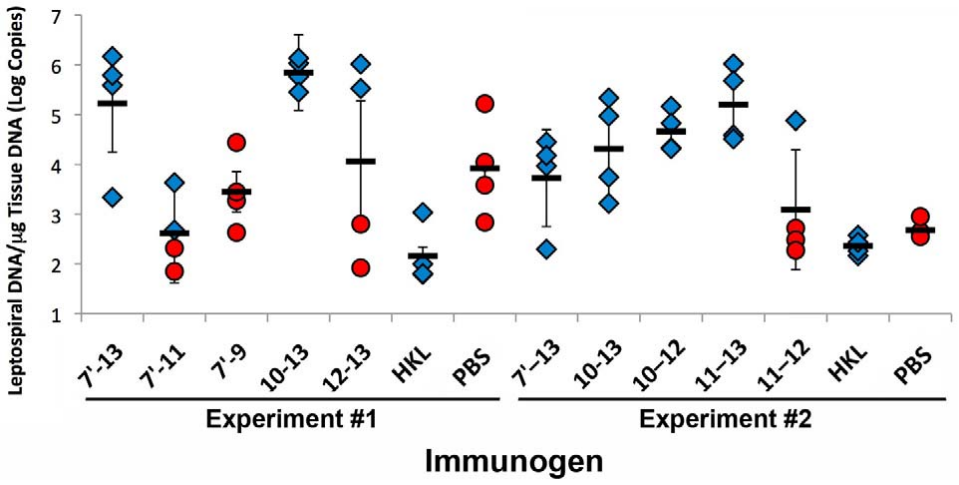
Leptospiral burden in kidney tissue. Kidney tissue was subjected to DNA extraction and real-time PCR to measure the leptospiral burden per microgram of tissue DNA. Means are depicted as bold horizontal bars along with standard deviations. Animals that survived to 28 days (blue diamonds) had higher leptospiral burdens than those that met end-point criterion early (red circles). Of the groups with 100% survival to 28 days, animals immunized with heat-killed leptospires (HKL) had lower bacterial burdens than those immunized with LigA fragments. LigA-immunized groups that survived up to 28 days were used for statistical comparisons (one-way ANOVA, P<0.05).

To more accurately assess the leptospiral burden, DNA from kidneys was analyzed by qPCR. As shown in Table 2 and Figure 5, groups immunized with LigA fragments had a mean of 1.2×10^3^ to 8×10^5^ copies of leptospiral DNA per microgram of kidney DNA. As expected, kidneys from animals immunized with heat-killed leptospires had a lower leptospiral burden than groups immunized with LigA proteins such as LigA10-12, LigA11-13, LigA10-13 (experiment #1) and LigA7′-13 (experiment #1) (non-parametric ANOVA, Dunńs post-test, P<0.05). Leptospiral burden appeared to have a significant effect on animal health as reflected in the weight of surviving hamsters; there was an inverse correlation (Pearson correlation coefficient -0.51, P<0.05) in experiment #2 between the percent weight gain during the last week of the experiment and the copies of leptospiral DNA per μg of kidney tissue DNA. However, there were no significant differences in the leptospiral burden among groups with 100% survival immunized with different LigA proteins (Non-parametric ANOVA, P>0.05).

### Pathology

**Figure 6:**
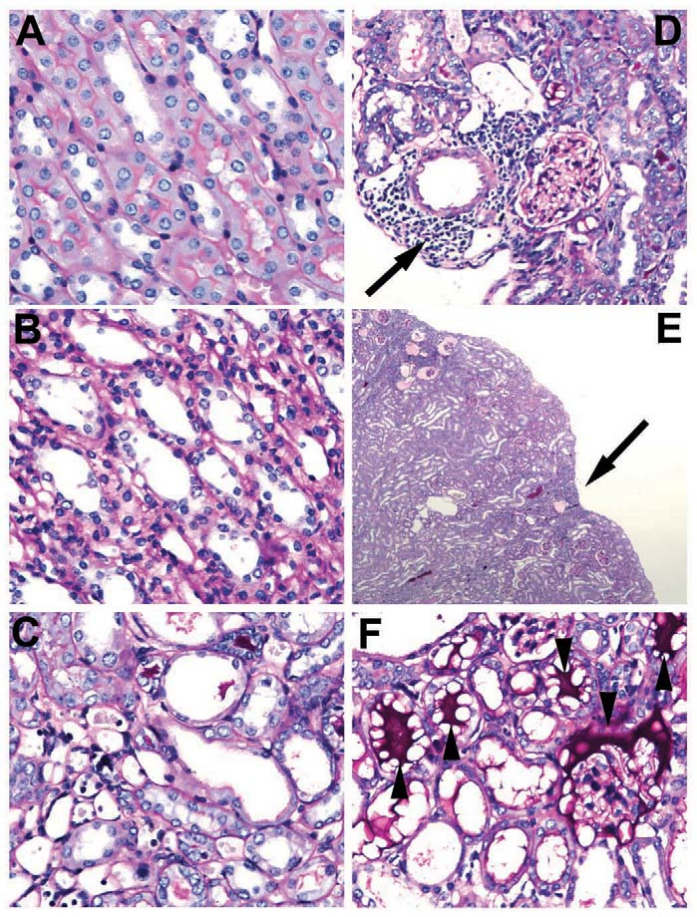
Renal histopathology showing tubulointerstitial changes. Representative PAS-stained kidney sections obtained from hamsters 28 days after leptospiral challenge showing, A. Normal tubular epithelium (40x); B. Moderate tubular damage (40x); C. Severe tubular atrophy (40x); D. Interstitial inflammation (arrow, 40x); E. Tubular scarring with depressed renal capsule (arrow, 4x); and F. Tubular deposition of intensely PAS-positive material consistent with Tamm-Horsfall glycoprotein (arrowheads, 40x).

Hemorrhagic areas were frequently noted on gross examination of the kidney and lungs of animals that did not survive challenge. Organs of survivors were usually normal in appearance but the kidneys occasionally appeared shrunken, pale, or had surface depressions indicating underlying infarction. Histopathological changes in the kidneys were largely limited to tubulointerstitial damage. Glomeruli were uniformly unaffected, except for one case of hyaline deposition seen in an HKL-immunized hamster. Although Bowman's space was dilated in some cases, the cells of the glomerulus were unaffected. Tubulointerstitial changes included renal tubular damage, encompassing changes of thinning of renal tubular epithelial cells (compare Figures 6A and 6B), increasing hyaline cast deposition, mitosis, tubular atrophy (Figure 6C), interstitial inflammation (Figure 6D), and associated capsular retraction (Figure 6E). Renal tubular obstruction was the most likely cause of hyaline cast deposition of the material staining intensely with PAS (Figure 6F). Other changes due to tubular obstruction were dilated Bowman's space with or without hyaline casts. Mitoses were seen in only 2 cases, which further supported tubular injury because the rate of tubular cell turnover is normally close to zero. As shown in Table 2, scores based on the extent of renal tubular damage were higher in groups immunized with the LigA10-12 and LigA11-12 proteins, suggesting that immunization with these constructs was associated with relatively more histopathology than other LigA constructs. Groups immunized with HKL and the LigA7′-13 protein had lower renal histopathology scores (Table 2) and there was an inverse correlation between renal histopathology score and weight gain (Pearson correlation coefficient -0.75, P<0.01). There was also an inverse correlation between renal histopathology score and leptospiral burden (Pearson correlation coefficient −0.84, P<0.01) for animals with>1.5×10^4^ copies of leptospiral DNA/µg of tissue DNA, suggesting that a more intense immune response (reflected by interstitial nephritis) may be partially effective at clearing residual infection.

Serum chemistries were measured to evaluate liver and kidney function of the hamsters (Table 3). Alanine aminotransferase and alkaline phosphatase levels were moderately elevated in all groups, consistent with hepatitis and cholestasis, respectively. However, bilirubin levels were universally normal, indicating that hepatic cholestasis had not progressed to biliary obstruction. Blood urea nitrogen (BUN) levels were increased in all groups and extremely elevated in the PBS control, while creatinine was low in all groups and elevated in the PBS control group (one-way ANOVA with Dunn's post test, P<0.05), indicating that renal dysfunction and/or dehydration contributed to mortality in these animals. In contrast, serum creatinine and BUN levels were universally normal in survivors, indicating that the renal tubular damage observed by histopathology had not progressed to frank kidney failure.

**Table 3:** Chemistry results^a^.

Group(LigA Protein)	BUN^b^(mg/dL)	AlkPhos^b^(IU/L)	Calcium(mg/dL)	Creatinine(mg/dL)	Phos^b^(mg/dL)	SGPT^b^(IU/L)	Total bilirubin (mg/dL)	Total protein (g/dL)
7′-13	21±2	**127**±8*	13±0.7	0.3±0.1	7.6±0.5	64±11	0.1±0	6.6±0.4
10-13	25±5	78±14	12.8±2.2	0.2±0	6.8±1.5	57±26	0.1±0	5.6±1
10-12	26±6	72±4	11.6±1.8	0.2±0	6.6±1	39±20	0.1±0	5.3±0.6
11-12	67 ±77	71±18	9.7±1.3	0.8±1.2	7.5 ± 3.3	49±10	0.1±0	**4.6**±**0.6**
11-13	22±2	**98**±**4**	13±0.9	0.2±0.1	7.6±0.6	51±16	0.1±0	6.2±0.5
HKL^c^	23±2	73±10	12.5±0.9	0.2±0	6.8±1	42±2	0.1±0	6.2±0.5
PBS^d^	**235**±49*	**130**±15*	13.7±1.4	**6.8**±1.6*	18.1±1	48±5	0.3±0.1	6.7±0.4
Ref. range	9–30	15–45	8–12	0.5–2.2	4.2–8.5	10–35	0–1	4.5–6.5

a Means with standard deviations from Experiment 2 are displayed (n = 4). Bold numbers: Significantly different from group immunized with heat-killed leptospires (one-way ANOVA with Dunn's post test, P<0.05). *Significantly different from group immunized with heat-killed leptospires (one-way ANOVA with Dunn's post test, P<0.01).

b Abbreviations: BUN, blood urea nitrogen; AlkPhos, alkaline phosphatase; Phos, phosphorus; SGPT, serum glutamic pyruvic transaminase.

c Heat-killed leptospires.

d Phosphate buffered saline.

## Discussion

In this study, we identified the LigA domains involved in protecting hamsters from lethal leptospiral infection. Intraperitoneal inoculation was performed with 1000 *L. interrogans* serovar Copenhageni strain Fiocruz L1-130, resulting in a lethal infection in all control animals (Table 2, Figure 3). This is the same challenge dose used in a previously successful LigA protection study and is estimated to be ∼20-fold over the LD_50_ for this strain [27]. We found that a LigA protein construct consisting of at least three Big domains is required for immunoprotection and that the 11^th^ and 12^th^ specific Big domains must be included in this construct. Given that the average pairwise sequence identity among LigA Big domains is only 37% [32], the domains identified here are likely to be antigenically unique and contain unique immunoprotective epitopes. Compared to maximally protective proteins, less protective LigA proteins elicited similar antibody titers in hamsters (Figure 1), suggesting that protection was not solely due to the antigenicity of the respective LigA vaccine. The mechanism of LigA mediated immunoprotection has not been elucidated, but may involve the disruption of a key function of LigA in leptospiral pathogenesis and/or the enhancement of host defense mechanisms. One key function of LigA is to mediate binding of *Leptospira* to host molecules such as fibronectin and fibrinogen [24]. Fibronectin- and fibrinogen-binding activity is found within domains 7 through 13 of LigA, with the carboxy-proximal domains 10 to 13 being required for fibronectin binding (unpublished study, H. A. Choy). Finer mapping of the LigA binding activities may give clues as to the possible immunoprotective mechanism.

As noted previously, LigA immunization converts an otherwise lethal infection into a sublethal kidney infection [27]. The burden of infection and its effects on vaccinated hamsters, qPCR and a histopathology scoring system were included as quantitative outcome measures. To our knowledge, this is the first vaccine study to use qPCR to quantitate leptospiral burden in animals after challenge. The application of qPCR to leptospiral vaccine studies allows for the accurate determination of the leptospiral burden, especially in the kidney, where colonization can lead to kidney damage and/or urinary shedding of the pathogen. We found that the heat-killed leptospires may not confer sterilizing immunity. Although the kidneys from the immunized animals were culture negative, leptospiral DNA was detected by qPCR. Reverse transcription-qPCR studies are needed to determine whether the low levels of DNA in these kidneys represent viable spirochetes or are remnants of leptospires killed by the host immune system. Comparison of quantitation results among surviving hamsters shows that immunization with as few as three LigA domains did not result in significantly higher levels of renal colonization than immunization with longer constructs such as the seven-domain LigA7′-13 protein (Figure 5). However, immunization with LigA10-12 did lead to greater histopathology, indicating different protective effects of the LigA10-12 and LigA11-13 constructs (Table 2).

Histopathology analysis of kidney sections was performed using PAS staining, which is useful for evaluating many different types of nephropathology, including the severity of tubulointerstitial damage in our study. PAS staining facilitated identification of proximal tubules by their carbohydrate-containing brush border, evaluation of tubular basement membrane changes, as well as tubular atrophy (Figures 6A, B, and C). A striking finding of our study was the identification of intensely staining protein casts in the tubules of 32% of animals, both in solid and “bubbly” deposition patterns (Figure 6E). These protein casts probably represent Tamm-Horsfall glycoprotein (THP), also known as uromodulin or TAMM protein, a glycoprotein that is produced by renal tubular epithelial cells [39]. THP is the most abundant protein in mammalian urine and though its deposition, in and of itself, is not pathologic, the high frequency of THP deposition in our study, including one case with extensive tubular deposition that occurred in an animal that succumbed to acute leptospirosis, suggests that increased THP deposition is related to the pathogenesis of leptospiral renal pathology. These physiologic hyaline deposits are usually solid, but in our study all cases demonstrated both a solid and “bubbly” deposition pattern. This “bubbly” pattern appeared to be due to a pathological process rather than an artifact of fixation and/or embedding, but further studies are needed to confirm this conclusion.

Insufficient information is currently available to understand how broadly LigA immunoprotection can be applied. Whereas *ligB* has been found in all pathogenic *Leptospira* species, *ligA* has been found in only *L. interrogans* and *L. kirschneri* [32]. *L. interrogans* serovar Lai is the only *L. interrogans* isolate found not to contain *ligA* [40]. If *ligA* deficiency is confirmed in other Lai isolates, this would be a notable exception because the organism is both highly virulent and epidemiologically important. Recently, it was reported that homologous immunization with LigA7-13 that was expressed and purified under denaturing conditions did not protect hamsters against lethal infection by *L. interrogans* serovar Manilae strain L495, an organism that expresses LigA [19]. This result stands in stark contrast to previously successful immunization studies involving *L. interrogans* serovars Manilae (strain UP-MMC-NIID), Copenhageni and Pomona [27], [28], [41]. Although there were differences in the strains and adjuvants used, the finding that denatured LigA did not protect against lethal challenge could indicate that the protective epitope is conformational rather than linear. Accordingly, our finding that protective segments include domains 11 and 12 plus a third domain (10 or 13) on either end, suggests that three domains may be required for proper conformational folding. Additional research is needed to further define the structural requirements for LigA vaccine efficacy.

We strongly recommend daily weighing of animals in leptospiral challenge experiments, including studies evaluating vaccine efficacy. We found that 10% weight loss effectively identified animals with leptospiral infection that had advanced to a premorbid condition. A similar result was observed in a recent study of leptospirosis in guinea pigs [42]. Weight loss is an objective end-point criterion that avoids uncertainty about whether an animal is able to eat and drink sufficient amounts of food and water. Thus, weight should be monitored along with other clinical parameters as different challenge doses or different strains may not present the same pattern of disease.

In summary, we have mapped the immunoprotective segment of LigA and determined the minimal number of domains necessary to protect hamsters from lethal infection. This work also extends previous studies by quantifying the sublethal burden of infection and by defining the renal histopathological consequences of infection. It is worth noting that the immunoprotective domains we identified are contained within a segment that is known to mediate interactions with host extracellular matrix proteins [24]. This suggests that LigA-mediated immunoprotection may involve interference with key leptospiral-host interactions rather than a bactericidal mechanism. Further studies to define the kinetics of leptospiral infection in immunized animals may provide insight into both the mechanism of LigA-mediated immunoprotection and the development of vaccines for sterilizing immunity against leptospirosis.
